# Complete Genome Sequence of Helicobacter suis Strain SNTW101c, Originally Isolated from a Patient with Nodular Gastritis

**DOI:** 10.1128/MRA.01340-19

**Published:** 2020-01-02

**Authors:** Emiko Rimbara, Masato Suzuki, Hidenori Matsui, Masahiko Nakamura, Hirotaka Kobayashi, Shigetarou Mori, Keigo Shibayama

**Affiliations:** a Department of Bacteriology II, National Institute of Infectious Diseases, Tokyo, Japan; b Antimicrobial Resistance Research Center, National Institute of Infectious Diseases, Tokyo, Japan; c Kitasato Institute for Life Sciences, Kitasato University, Tokyo, Japan; d Graduate School of Control Sciences, Kitasato University, Tokyo, Japan; e School of Pharmaceutical Sciences, Kitasato University, Tokyo, Japan; f Department of Pathology, National Institute of Infectious Diseases, Tokyo, Japan; University of Rochester School of Medicine and Dentistry

## Abstract

Helicobacter suis strain SNTW101c, which was originally obtained from a patient with nodular gastritis, has been maintained in mouse stomach because of difficulty culturing it *in vitro*. Recently, we succeeded in culturing this strain *in vitro*. Here, we report the complete genome sequence of H. suis strain SNTW101c.

## ANNOUNCEMENT

Helicobacter suis frequently colonizes pig stomach and causes gastric diseases, including mucosa-associated lymphoid tissue (MALT) lymphoma, in humans (1). H. suis strain SNTW101 was originally isolated from a patient with nodular gastritis in 2008; thereafter, it has been passaged in mice because the bacteria could not be grown *in vitro*. We previously reported a draft genome sequence of strain SNTW101 that was determined using bacterial cells purified from SNTW101-colonized mouse stomach with anti-Helicobacter pylori antibody-coated magnetic beads (2). Recently, the bacteria were successfully isolated from the infected stomach and grown *in vitro* by a method described previously (3, 4). Here, we report the complete genome sequence of the resulting H. suis strain, named SNTW101c.

**FIG 1:**
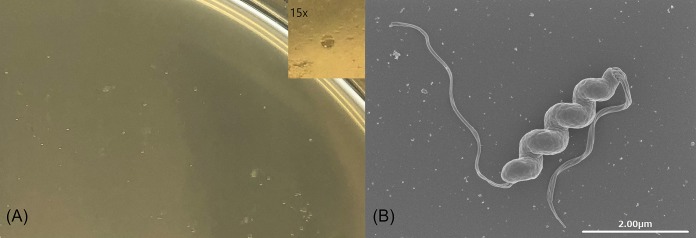
Helicobacter suis strain SNTW101c: (A) colonies on an agar plate; (B) scanning electron microscopy image.

SNTW101c was isolated as described previously (3, 4). The colonies appeared 20 days after inoculation (Fig. 1A), and each single colony was further subcultured in a biphasic medium containing *Brucella* broth and agar with Vitox and Skirrow supplements (Thermo Fisher Scientific), 0.05% HCl, and 20% fetal bovine serum (Sigma-Aldrich). The morphology of H. suis SNTW101c included a tightly coiled body with sheathed bipolar flagella (Fig. 1B), which contribute to the high level of motility of these bacteria (see https://youtu.be/l70zI-9N74A). A DNA library was prepared, using a rapid barcoding kit (product number SQK-RBK004; Oxford Nanopore Technologies), from genomic DNA extracted using Qiagen Genomic-tips 20/G and buffers (Qiagen). Nanopore sequencing using the MinION platform with R9.4.1 flow cells (Oxford Nanopore Technologies) provided a total of 11,701 reads (*N*_50_ = 11,501 bp), with an average coverage depth of 26.7; Guppy v3.1.5 (Oxford Nanopore Technologies) was used for base calling and adapter trimming, with default parameters. *De novo* assembly was performed with Unicycler v0.4.8 (5), with default parameters, and three contigs, including one chromosome and two putative plasmids, were constructed. The overlap region in the assembled contig was detected by a genome-scale sequence comparison using LAST (http://last.cbrc.jp) and was trimmed manually. The genomic DNA was also sequenced on an Illumina MiniSeq system, with a MiniSeq high-output reagent kit (300 cycles), using a 151-bp paired-end library prepared with the Nextera XT DNA library preparation kit (insert size, 500 to 900 bp); this resulted in a total of 263,361 reads (*N*_50_ = 151 bp) and an average coverage depth of 46.1. Illumina reads were mapped onto the sequences assembled *de novo* from MinION reads, and sequencing errors were corrected by extracting the consensus of the mapped reads three times using CLC Genomics Workbench v11.0.1 (Qiagen), with default parameters. The resulting sequences were annotated using DFAST v1.1.0 (6), with default parameters. The genome size of SNTW101c was 1,680,021 bp, comprising 1,744 protein-coding sequences (CDSs) and 5 ribosomal RNAs, with a GC content of 40%. The genome size of SNTW101c was similar to that estimated for SNTW101 (1,608,632 bp) (2). The putative plasmids pSNTW101c_1 (9,051 bp) and pSNTW101c_2 (5,825 bp) coded for 6 CDSs each.

As suggested by previous studies (1, 7), both CagA and VacA, the major virulence factors of the human gastric pathogen Helicobacter pylori (8), were absent in strain SNTW101c, indicating that unknown virulence factors contribute to bacterial pathogenesis. Regarding the plasmids, pSNTW101c_1 possessed genes encoding a type IIS restriction-modification system.

This is the first report of the complete genome sequence of an H. suis strain and will aid in understanding the mechanism of chronic H. suis infection in the stomach and bacterial pathogenesis associated with MALT lymphoma in humans.

### Data availability.

The complete genome sequence of H. suis strain SNTW101c was deposited in NCBI GenBank under the accession numbers AP019774 (chromosome), AP019775 (pSNTW101c_1), and AP019776 (pSNTW101c_2). The raw sequence data are available in the Sequence Read Archive with the accession numbers DRX176097 (Illumina) and DRX176341 (MinION).
